# 

**Published:** 1885-09

**Authors:** 


					﻿Vhole Number, iOdr
SEPTEMBER, 1885
Vol. LI., Number 3
TZE32ZED
CHICAGO MEDICAL JOURNAL AND EXAMINER
EDITORS:
S. J. JONES, M.D., LL.D.
W. W. JAGGARD, A.M., M.D.
HAROLD N MOYER, M D
CHICAGO
PUBLISHED MONTHLY BY THE CHICAGO MEDICAL PRESS ASSOCIATION
240 Wabash Avenue.
CLARK & LONGLEY. PRINTERS. 183 AND 183 DEARBORN STREET, CHICAGO.
				

